# Integrated Mapping of Yaws and Trachoma in the Five Northern-Most Provinces of Vanuatu

**DOI:** 10.1371/journal.pntd.0005267

**Published:** 2017-01-24

**Authors:** Fasihah Taleo, Colin K Macleod, Michael Marks, Oliver Sokana, Anna Last, Rebecca Willis, Mackline Garae, Annie Bong, Brian K Chu, Paul Courtright, Jacob Kool, George Taleo, Jean Jacque Rory, Anthony W Solomon, Global Trachoma Mapping Project

**Affiliations:** 1 Ministry of Health, Port Vila, Vanuatu; 2 Clinical Research Department, London School of Hygiene & Tropical Medicine, London, United Kingdom; 3 Sightsavers, Haywards Heath, United Kingdom; 4 Hospital for Tropical Diseases, London, United Kingdom; 5 Eyecare Department, Ministry of Health, Honiara, Solomon Islands; 6 Task Force for Global Health, Decatur, GA, United States of America; 7 Ophthalmology Department, Port Vila Central Hospital, Port Vila, Vanuatu; 8 KCCO International, Division of Ophthalmology, University of Cape Town, Cape Town, South Africa; 9 WHO Country Office, World Health Organization, Port Vila, Vanuatu; 10 Department of Control of Neglected Tropical Diseases, World Health Organization, Geneva, Switzerland; University of Washington, UNITED STATES

## Abstract

Yaws and trachoma are targeted for eradication and elimination as public health problems. In trachoma-endemic populations mass administration of azithromycin can simultaneously treat yaws. We conducted a population-based prevalence survey in the five northernmost provinces of Vanuatu, where trachoma and yaws are suspected to be co-endemic. Clinical signs of trachoma were evaluated using the WHO simplified grading system, and skin examination with a serological rapid diagnostic test used to identify yaws. We enrolled 1004 households in 59 villages over 16 islands, and examined 3650 individuals of all ages for trachoma. The overall adjusted prevalence of trachomatous inflammation-follicular (TF) in 1–9 year-olds was 12.0% (95% Confidence Interval: 8.1–16.7%), and the overall adjusted prevalence of TT in those aged 15 years and greater was 0.04% (95% CI 0–0.14%). In multivariate analysis, the odds of children having TF was 2.6 (95% CI = 1.5–4.4) times higher in households with unimproved latrines, and independently associated with the number of children in the household (OR 1.3, 95% CI = 1.0–1.6 for each additional child). We examined the skin of 821 children aged 5–14 years. Two children had yaws, giving an estimated prevalence of active yaws in those aged 5–14 years of 0.2% (95% CI = 0.03–0.9%). Mass treatment with azithromycin is recommended in these provinces. Given the apparent low burden of yaws, integration of yaws and trachoma control programmes is likely to be useful and cost-effective to national programmes.

## Author Summary

Yaws and trachoma are infectious diseases targeted by the World Health Organization (WHO) for eradication and elimination as a public health problem, respectively. Both diseases are found in the rural, isolated and underserved communities of the world’s poorest countries. The WHO strategy for trachoma elimination includes Mass Drug Administration (MDA) of the antibiotic azithromycin, with population-based prevalence surveys required to determine where interventions are needed. As yaws can also be treated with azithromycin, in co-endemic areas, azithromycin MDA will likely treat both infections. We conducted an integrated trachoma and yaws prevalence survey in establish the prevalence of both diseases in the 5 northern-most provinces of Vanuatu. The estimated prevalence of trachomatous inflammation—follicular (TF, a sign of *C*. *trachomatis* infection) in children aged 1–9 years was 12.0%, and the estimated prevalence of trachomatous trichiasis (TT, advanced, potentially blinding disease) in adults aged 15 years and above was 0.04%. Two children had yaws, giving an estimated prevalence of active yaws in those aged 5–14 years of 0.2%. Mass treatment with azithromycin is recommended in these provinces. Given the apparent low burden of yaws, integration of yaws and trachoma control programmes is likely to be useful and cost-effective to national programmes.

## Introduction

The World Health Assembly has targeted yaws and trachoma for global eradication and elimination-as-a-public-health-problem, respectively, by the year 2020[1]. Both are neglected tropical diseases found in rural areas of the world’s poorest countries. Trachoma is the world’s most common infectious cause of blindness, with an estimated 232 million people at risk of irreversible blindness in 51 countries[2]. Yaws is an infectious disease that causes disfiguring and often painful lesions of the skin and bones. Although data are limited, it is thought to be endemic in at least 13 countries, including three in the Pacific[3].

Trachoma is caused by ocular infection with the bacterium *Chlamydia trachomatis*. Infection is spread by direct or indirect contact[4][5], and is linked to poor sanitation, crowded living conditions and inadequate access to water[6]. Infection in children is self-limiting and may present with lymphoid follicles on the upper tarsal conjunctivae, known as trachomatous inflammation-follicular (TF), and/or intense inflammatory thickening of the conjunctivae, known as trachomatous inflammation-intense (TI). Recurrent infection can lead to scarring of the tarsal conjunctivae and changes in the morphology of the eyelid, leading to trachomatous trichiasis (TT), where the eyelashes turn inwards and rub on the globe. Over time, abrasion of the cornea by eyelashes can lead to an irreversible opacification of the normally clear cornea that may impair vision and eventually lead to blindness [7][8].

Yaws is caused by infection with the bacterium *Treponema pallidum* subsp. *pertenue*. It is transmitted from person to person through direct skin contact. It presents with papillomas or normally painless ulcers which, if left untreated, may be followed by multiple skin lesions and sometimes more severe tissue and bone disease. The majority of clinical cases are seen in children under 15 years old (peak incidence 2–10 years), predominantly in isolated rural areas that have warm and humid climates [9].

The latest WHO clinical guidelines recommend that a confirmed case of yaws should lead to treatment of the entire community[10]. Implementation of these are challenging if health workers do not have a means to confirm suspected yaws cases. Traditional syphilis serology combines a highly specific treponemal antibody test (TPPA/TPHA) with a less-specific non-treponemal antibody test (VDRL/RPR). The former test remains positive for life, whilst the latter varies over time, more accurately reflecting current disease activity. A reliable rapid diagnostic test (RDT) (DPP-Syphilis Screen and Confirm Chembio, USA) [11][12] has been developed with a sensitivity and specificity for treponemal antibodies of 88% and 95% respectively and for non-treponemal antibodies of 88% and 93% respectively.

Mass treatment with azithromycin forms part of the WHO strategies for the elimination of trachoma and the eradication of yaws. In co-endemic regions, MDA with azithromycin reduces the community prevalence of both infections[13,14], and therefore the integration of surveys for the two diseases is a logical step to provide baseline data for treatment planning.

**Fig 1:**
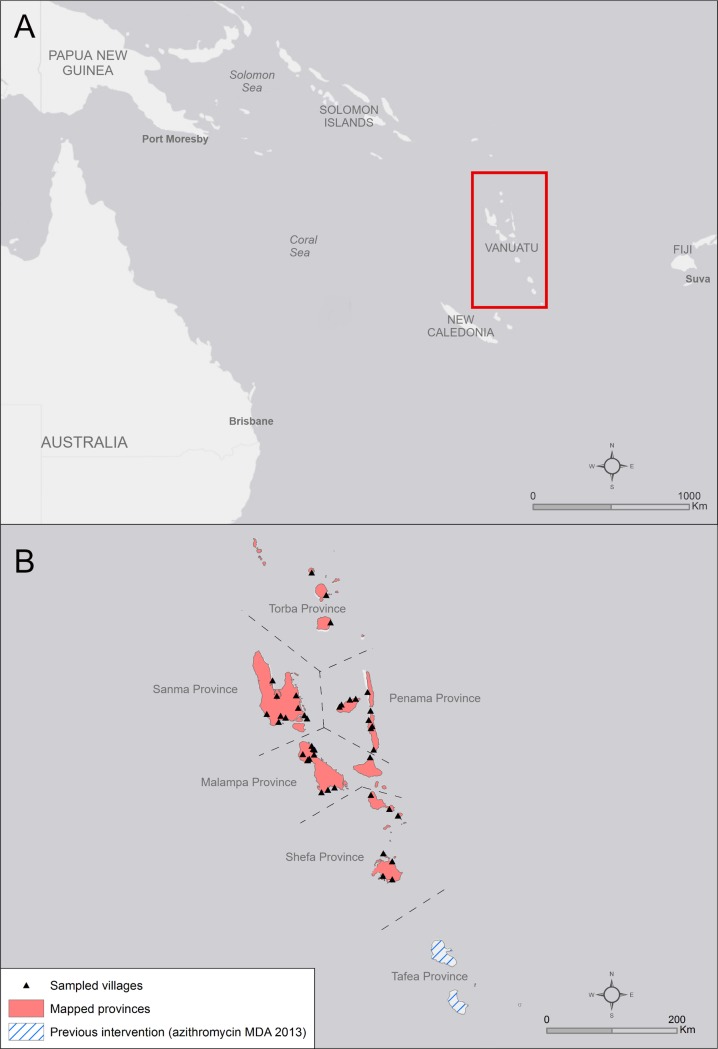
(Panel A) General location map for Vanuatu. (Panel B) Provinces and villages over 12 islands included in the survey, Global Trachoma Mapping Project, Vanuatu, 2014. Shapefile credit: Global Administrative Areas (gadm.org); Basemap credit: Esri, Openstreetmap.

Vanuatu is a country comprising approximately 80 islands, situated approximately 1800km North-East of Australia (Fig 1 (Panel A)). It has a total population of over 230,000 people and is composed of 6 provinces: Torba, Sanma, Penama, Malampa, Shefa and Tafea (Fig 1 (Panel B)) [15].

In 2009, a trachoma rapid assessment was conducted in 17 sites in Tafea and Shefa province, indicating that trachoma was a likely to be significant public health burden [16]. A population-based prevalence survey was therefore required to determine the prevalence of TF in Vanuatu, to guide application of community-level interventions[17].

In 2011 and 2012, the Vanuatu government reported 2,197 and 2,154 cases of yaws, respectively[18], with the highest reported numbers of cases occurring in the Provinces of Tafea and Sanma. In 2013, as part of the national yaws control programme, the government carried out mass drug administration (MDA) of azithromycin in Tafea province.

We carried out a cross-sectional prevalence survey in order to estimate the baseline prevalence of trachoma and yaws in the 5 northern-most provinces of Vanuatu. The objectives were to estimate the prevalence of TF in children aged 1–9 years, the prevalence of TT in adults aged 15 years and greater, and the prevalence of yaws skin lesions in children aged 5–14 years. Due to the recent MDA with azithromycin, Tafea province was excluded from these surveys.

## Methods

A two-stage cluster-randomised survey was conducted between 20^th^ October to the 21^st^ November 2014, and followed the standard methodological principles of the Global Trachoma Mapping Project (GTMP)[19]. Excluding Tafea Province, the provinces of Vanuatu surveyed have a total estimated rural population of 144,276[20] and were therefore considered as one Evaluation Unit (EU), in accordance with WHO guidelines for trachoma prevalence surveys [21]. Based on the anticipated number of children per household from the latest census data, 42 clusters were selected for survey. The islands were stratified in the sampling to ensure representation from each island group. Clusters were selected from island groups using probability proportional to size methodology. In each selected cluster, 30 households were selected randomly by drawing lots from a list of names of household heads compiled at the time of survey. If selected clusters contained fewer than 30 households, all households were invited to participate, and a number of randomly selected households from the next-nearest village sufficient to bring the total to 30 were randomly selected using the same methods.

After obtaining informed consent, all individuals over the age of one year from selected households were examined for clinical signs of trachoma using the WHO simplified grading system[8][19]. Additionally, in those aged 5–14 years, graders performed an examination of the skin of the whole body, excluding the genitals and buttocks. Prior to the survey, graders were trained to perform a dermatology exam to recognise skin lesions consistent with active yaws, as classified in the WHO yaws pictorial guide [22]. In children with skin lesions consistent with active yaws, a finger-prick blood sample was collected and an RDT for yaws was performed. The RDT was considered positive if both the treponemal and non-treponemal component were positive in accordance with manufacturer’s instructions.

## Household Risk factors Survey

Data collectors were trained to collect Water, Sanitation and Hygiene (WASH) variables at household level[19]. Data were obtained by interviewing the head of the household or responsible adult. The questionnaire included items on access to and use of latrines, access to and use of water sources for drinking and washing, and the estimated time needed to retrieve water. The questionnaire was supported through use of observational data obtained by the data collectors on latrine type, handwashing facilities near to the latrine and the presence of soap and water at the handwashing facility at the time of survey.

## Statistical Analysis

Statistical analysis for risk factors was carried out using Stata 10.2 (Stata Corp, TX,USA). Confidence intervals around prevalence estimates were constructed by bootstrapping the adjusted cluster proportions for TF and yaws in R 3.0.2 (The R Foundation for Statistical Computing, 2013). An exact binomial confidence interval was used to derive a TT upper confidence interval. Risk factor analysis was restricted to the binary outcome of the presence of TF in 1–9 year old children. Where present, TF is maximally found in this age-group, and control interventions are tiered by the prevalence in this age range. A mixed effects logistic regression model was used to evaluate the effect of each explanatory variable. Odds ratios were adjusted by household and cluster. Univariable associations were included in the multivariable model if p≤0.05. Mantel-Haenszel Chi-square tests were used to assess collinearity between independent variables, with variables considered for exclusion if significant collinearity was demonstrated (p≤0.05). A multivariable model was developed using a step-wise inclusion approach, with the decision to retain a variable based on a likelihood ratio test between the included/excluded models using a significance level of p = 0.05. Age and sex were included in the model *a priori*.

## Ethical Considerations

The overall study protocol was approved by the Executive Committee of the Ministry of Health of Vanuatu, and the ethics committee at the London School of Hygiene & Tropical Medicine. Both ethics committees considered the project to be a low-risk activity considered to be part of routine public health services, and therefore approved the use of verbal consent throughout the project.

All adult subjects provided informed consent before examination. For all those examined under 18 years of age, consent was provided by their parent or guardian on their behalf. Consent was recorded electronically on a custom-made smartphone application used for all data collection.

All individuals found to have TF or TI were offered treatment with oral azithromycin or topical tetracycline. All individuals found to have signs of TT were referred for further assessment at the nearest eyecare centre. All individuals found to have clinical evidence of active yaws were offered treatment with azithromycin. In addition, all villages where cases of yaws were found were offered total community treatment with azithromycin. All data were anonymised after collection for the purposes of analysis.

## Results

A total of 59 villages were surveyed over 16 islands. We invited 1004 households and 4095 individuals to participate, resulting 3650 (89.1%) individuals consenting to examination (S1 Table). 1869 (51.2%) of those examined were female. 240 (5.8%) household members were absent at the time of survey and 205 (5.0%) did not give consent to examination.

135(14.5%) of 928 children aged 1–9 years had TF, with an age-adjusted TF prevalence of 12.0% (95%CI 8.1–16.7) Only two cases of TT were identified out of 2511 adults aged 15 years and greater. This represents an age- and sex-adjusted TT prevalence in adults of 0.04% (95%CI 0.0–0.14%; unadjusted prevalence in adults 0.08%). 3 additional TT cases were identified in children aged 5, 6 and 14 years, which, after referral for ophthalmology opinion, were found to be false positives. The adjusted prevalence of TT in the whole population was 0.02% (95%CI 0–0.1%; unadjusted prevalence 0.04%)

## Clinical Yaws

1000 children aged 5–14 years were sampled for inclusion in the study. 821 (82.1%) consented to examination. Twenty-two (2.7%) had clinical signs consistent with yaws. Six children (0.7%) had skin lesions consistent with yaws–three had papillomata, two had ulcers, one had squamous macules. A total of 18 children (2.2%) had objective bony swelling on examination, with 16 having no associated skin lesions. Two children (0.2%) met the criteria for clinically active yaws, having skin lesions consistent with yaws and objective bony swelling.

## Treponemal RDT Results

Five children with skin lesions consistent with yaws and had a subsequent RDT. Only 2 out of 5 children had a positive RDT. Of the two children with both skin lesions and bony swelling one had a positive RDT. Three children were reported to have had treatment for yaws with benzathine penicillin in the preceding 12 months. The estimated population-level prevalence of active yaws in 5–14 years was 0.2% (95%CI 0.03–0.9%).

## Risk Factors Associated with TF

Univariable analyses of associations of TF are shown in S2 Table. All children included in the study had access to a shared or private latrine. The final multivariable model (S3 Table) showed that TF was strongly and independently associated with a household’s use of an unimproved pit latrine (OR 2.6 (95%CI 1.5–4.4)), the number of children in the household (OR 1.3 –linear increase with each additional child (95%CI 1.0–1.6)), and a child’s age (OR 1.1 –linear increase with each additional year (95%CI 1.0–1.2)).

## Discussion

Trachoma is endemic in Vanuatu. The prevalence of TF in children aged 1–9 years exceeds the threshold above which the WHO recommends MDA with azithromycin as well as facial cleanliness and environmental improvement initiatives. The age and sex standardised TT prevalence showed that TT was not a significant public health problem, and was below the WHO elimination threshold of 0.2% of the population aged 15 years and greater[23].

Consistent with other studies, we found that the number of children living in the household aged 1–9 years was strongly associated with TF[24,25], the odds increasing with each additional child. Trachoma is thought to be spread through close contact, via exposure to infected ocular or nasal secretions. The reservoir of infection is predominantly in children[26–28], and so it stands to reason that the more close contact opportunities children have with other potentially-infected children, the higher the chance that they will become infected themselves. This association was not maintained when considering the number of inhabitants of the household overall, consistent with contact with infected children preferentially facilitating spread.

All households reported having access to some form of latrine and open air defaecation was not reportedly common. The WHO/UNICEF Joint Monitoring Programme (JMP) for Water Supply and Sanitation defines an unimproved form of sanitation to be that which fails to separate faecal material from human contact[29]. We found unimproved sanitation facilities in 385 households in which children aged 1–9 years were resident. After accounting for other possible confounders, children living in such households were 2.6 times more likely to have signs of TF in the survey.

Trachoma has previously been associated with poor sanitation [6,30,31]. This is thought to be because areas of open defecation provide a preferential breeding ground for eye-seeking *Musca sorbens* flies which can mechanically transmit *C*. *trachomatis* between individuals as a passive vector. *M*. *sorbens* preferentially breeds on human faeces left lying on the soil, with faeces in pit latrines of any kind, including “unimproved” latrines, not constituting a site for oviposition [32,33]. In our data, poor latrine access may simply be a surrogate for deprivation overall, with its associated limitations in economic and educational opportunities. It was not possible to control for these variables in our analyses.

In addition, any sanitation that is shared between households is considered to be unimproved. This is based on the belief that in shared facilities there are few incentives for individual users to keep the facility clean, and more vulnerable groups, including women and children, are less likely to use them[34,35]. In our survey the association between TF and the use of an unimproved latrine was not maintained in multivariable analyses if shared latrines alone were considered as a risk factor, suggesting that the latrine type available, rather than communal use conferred increased risk.

We found an increasing odds of TF with increasing age in the range 1–9 years. Most studies report a higher prevalence and odds of TF in younger children[27,36]. Superficially this may be at odds with suggestions that young children are the main reservoir of infection in this population, although we are careful here to acknowledge the difference between ocular *C*. *trachomatis* infection and the clinical signs of active trachoma. In highly endemic areas, infection is likely to be first acquired in early infancy, with the age-of-first-acquisition on average increasing with decreasing prevalence. These differences in the age associations of infection and active trachoma are thought to be explained by a degree of immunity acquired in childhood, and socio-behavioural factors linked to hygiene practices and close contacts which may lessen the inflammatory response to, without changing the risk of, infection. It is possible that non-chlamydial bacteria such as *Streptococcus pneumonia* and *Haemophilus influenzae* also contribute to the follicular phenotype in individuals previously sensitised to *C*. *trachomatis* [37,38]. The phenotype seen may also be a response to other *C*. *trachomatis* serovars.

Due to the small number of cases of active yaws in this study we did not conduct a risk-factor analysis for yaws, but poor sanitation and reduced access to hand-washing facilities was associated with an increased risk of yaws in a previous study in the Pacific [39]. This suggests that, in addition to MDA, the F&E components of a trachoma elimination programme may have additional synergistic benefits for yaws control.

Although few in number, this survey identified cases of active yaws in Vanuatu. The aim for the WHO yaws control programme is eradication, therefore the identification and treatment of such cases is important. Yaws is seasonal, with higher numbers during the rainy season[40]. Our survey was conducted at the end of the dry season/beginning of the rainy season, and it is therefore possible that we have underestimated the true burden of disease. Additionally, the rapid diagnostic test was not performed on 16 individuals who had bony swellings but no skin lesions. The finding of bony swellings in isolation is consistent with active yaws, but this clinical sign was considered too non-specific to proceed to RDT in this survey. It was anticipated that the inclusion of those with this sign alone would lead to a prohibitively large number of RDTs being required, although in hindsight this would have only required a total of 22 RDTs (children with either bony swellings or skin lesions or both)—a finding that may guide future clinical/RDT surveys. Only 40% of children with skin lesions clinically consistent with yaws had a positive RDT, in keeping with a study conducted in Tafea province in 2013, in which 35.5% of children with clinically suspected yaws had positive serology [41]. These findings suggest that relying only on clinical diagnosis of yaws is insufficient and efforts should be made to increase access to RDTs. Asymptomatic treponemal infection was not evaluated, so we do not have an estimate of the prevalence of latent yaws in this population. In survey data from other countries in the Pacific, there can be between 4–10 latent cases of yaws per active case found[3,39,42]. The routine clinical case notification rate (which does not require serological confirmation) from the five provinces included in this study is approximately 0.2 cases per 100 people, which is consistent with the results found in our survey (personal communication- Fasihah Taleo)

Based on these findings, mass treatment with azithromycin and implementation of the F&E components of the SAFE strategy (with the provision of improved latrines where needed) are recommended in northern Vanuatu to eliminate trachoma and eradicate yaws as public health problems. Given the apparent low burden of yaws, integration of the two control programmes is likely to be useful and cost-effective to national programmes.

## Supporting Information

S1 Table Demographic characteristics of sampled individuals in a trachoma and yaws survey, Global Trachoma Mapping Project, the five northern-most provinces of Vanuatu, 2014. (DOCX)

S2 Table Multilevel univariable random effects logistic regression analysis of factors associated with the presence of trachomatous inflammation-follicular(TF) in children aged 1–9 years, in the five northern-most provinces of Vanuatu, 2014. (DOCX)

S3 Table Factors independently associated with trachomatous inflammation-follicular(TF) in children aged 1–9 years from multi-level multivariable random effects logistic regression analysis. (DOCX)

S1 Appendix STROBE Checklist for observational studies. (DOCX)
