# Supplementary material for: Integrated Mapping of Yaws and Trachoma in the Five Northern-Most Provinces of Vanuatu
Source: PLoS Negl Trop Dis. 2017 Jan 24;11(1):e0005267. doi: 10.1371/journal.pntd.0005267 (PMC5261559; doi:10.1371/journal.pntd.0005267)
Supplement: S1 Table — (DOCX) [file pntd.0005267.s001.docx]

| **Age** | **Sex** | **Examined(%)** | **Absent(%)** | **Refused(%)** | **Unavailable^a^ (%)** | **Total** |
| --- | --- | --- | --- | --- | --- | --- |
| **1-9** | **M** | 485(81.5) | 28(4.7) | 70(11.8) | 12(2.0) | 595 |
|  | **F** | 443(77.8) | 41(7.2) | 83(14.6) | 2(0.4) | 569 |
| **10-14** | **M** | 187(86.6) | 24(11.1) | 5(2.3) | - | 216 |
|  | **F** | 196(89.9) | 16(7.3) | 6(2.8) | - | 218 |
| **14+** | **M** | 1107(90.4) | 91(7.4) | 27(2.2) | - | 1225 |
|  | **F** | 1232(95.8) | 40(3.1) | 14(1.1) | - | 1286 |
|  | **Total** | 3650(89.1) | 240(5.9) | 205(5.0) |  | 4095 |
| ^a^sleeping at time of visit and unable to be contacted later | | | | | | |
