# Supplementary material for: Integrated Mapping of Yaws and Trachoma in the Five Northern-Most Provinces of Vanuatu
Source: PLoS Negl Trop Dis. 2017 Jan 24;11(1):e0005267. doi: 10.1371/journal.pntd.0005267 (PMC5261559; doi:10.1371/journal.pntd.0005267)
Supplement: S2 Table — (DOCX) [file pntd.0005267.s002.docx]

| **Table 2: Multilevel univariable random effects logistic regression analysis of factors associated with the presence of trachomatous inflammation-follicular(TF)** **in children aged 1-9 years, in five provinces of Vanuatu, 2014** | | | | | | | |
| --- | --- | --- | --- | --- | --- | --- | --- |
| **Variable** | **No. examined** | **Prevalence (%)** | **Univariate** | | | | |
|  | **(n=928)** | **TF** | **OR** ^a^ | **95%CI** | | **p-value** ^b^ | |
| **Age** |  |  |  |  | |  | |
| *1* | *117* | *5.1* | 1.13 (1.0-1.2)  *(each additional year)* | | | **0.003** | |
| *2* | *119* | *10.1* |  |  |  |  |  |
| *3* | *110* | *16.4* |  |  |  |  |  |
| *4* | *108* | *13.9* |  |  |  |  |  |
| *5* | *100* | *20* |  |  |  |  |  |
| *6* | *113* | *12.4* |  |  |  |  |  |
| *7* | *102* | *17.7* |  |  |  |  |  |
| *8* | *89* | *16.9* |  |  |  |  |  |
| *9* | *70* | *24.3* |  |  |  |  |  |
| **No. of 1-9 year olds in household** | - | - |  |  | |  | |
| *1* | *331* | *12.4* | 1.23  (1.0-1.5)  *(each additional child)* | | | **0.064** | |
| *2* | *316* | *12.7* |  |  |  |  |  |
| *3* | *189* | *19.1* |  |  |  |  |  |
| *4* | *72* | *15.3* |  |  |  |  |  |
| *5* | *20* | *35.0* |  |  |  |  |  |
| **No. living in household** |  | *-* |  |  | |  | |
| *1-5* | *606* | *13.0* | 1.0(baseline) |  | |  | |
| *>5* | *322* | *17.4* | 1.3 | *(0.8-2.1)* | | 0.294 | |
| **Sex** |  |  |  |  | |  | |
| *M* | *485* | *14.0* | 1.0(baseline) | - | | - | |
| *F* | *443* | *15.1* | 1.1 | 0.8-1.7 | | 0.516 | |
| **Specific latrine type** |  |  |  |  | |  | |
| *Unimproved* ^Δ^ | *385* | *21.8* | 2.4 | 1.4-3.9 | | **0.001** | |
| *Improved ^Ω^* | *543* | *9.4* | 1.0(baseline) | - | | - | |
| **Communal latrine?** |  |  |  |  | |  | |
| *Shared* | *183* | *19.1* | 1.7 | 1.0-3.0 | | **0.074** | |
| *Private* | *745* | *13.4* | 1.0(baseline) | - | |  | |
| **Specific drink water source** |  |  |  |  | |  | |
| *Unimproved^†^* | 362 | 19.9 | 1.8 | 1.0-3.1 | | **0.044** | |
| *Improved*^Ѳ^ | 566 | 11.1 | - | - | |  | |
| **Time to drinking source** |  |  |  |  | |  | |
| *Water source in the yard* | *371* | *11.8* | 1.0(baseline) |  | | 0.310 | |
| *Less than 30 mins* | *462* | *16.5* | 1.2 | 0.7-2.1 | |  | |
| *Between 30 mins and 1 hr* | *79* | *17.7* | 2.4 | 1.0-5.9 | |  | |
| *More than 1hr* | *16* | *6.3* | 0.9 | 0.1-13.3 | |  | |
| **Source of washing water** |  |  |  |  | |  | |
| *Unimproved^†^* | 450 | *18.4* | 2.0 | 1.1-3.6 | | **0.020** | |
| *Improved*^Ѳ^ | *478* | *10.9* | 1.0(baseline) |  | |  | |
| **Time to washing source** |  |  |  |  | |  | |
| *Water source in yard* | *295* | *10.9* | 1.0(baseline) | - | | 0.621 | |
| *Less than 30 mins* | *443* | *15.1* | 1.0 | 0.5-2.0 | |  | |
| *Between 30mins and 1 hr* | *92* | *17.4* | 2.0 | 0.8-5.3 | |  | |
| *More than 1hr* | *8* | *12.5* | 2.3 | 0.2-36.0 | |  | |
| *All face-washing at source* | *90* | *21.1* | 1.1 | 0.4-3.3 | |  | |
|  | | | | | | | |
| ^a^ Unadjusted Odds Ratio from univariate two-level mixed effects logistic regression | | | | | | | |
| ^b^ p-value from Wald's test^;^ variables significant at the p<0.10 level were considered for the multivariate model (highlighted in bold) | | | | | | | |
| ^Ω^*Flush/pour flush to septic tank, Flush/pour flush to pit latrine, Flush/our to open drains, Ventilated improved pit latrine (VIP), Pit latrine with slab* | | | | | | | |
| *^Δ^ Pit latrine without slab/open pit, Hanging toilet/hanging latrine* | | |  | |  | |  |
| *^Ѳ^ Piped water into dwelling or yard/plot, public tap or standpipe, tubewell or borehole, protected dug well, protected spring, rainwater* | | |  | |  | |  |
| ^†^ Unprotected spring or dug well, surface water, bottled water | | |  | |  | |  |
