# Supplementary material for: Integrated Mapping of Yaws and Trachoma in the Five Northern-Most Provinces of Vanuatu
Source: PLoS Negl Trop Dis. 2017 Jan 24;11(1):e0005267. doi: 10.1371/journal.pntd.0005267 (PMC5261559; doi:10.1371/journal.pntd.0005267)
Supplement: S3 Table — (DOCX) [file pntd.0005267.s003.docx]

| **Table 3: Factors independently associated with trachomatous inflammation-follicular(TF) in children aged 1-9 years from multi-level multivariable random effects logistic regression analysis** | | | | |
| --- | --- | --- | --- | --- |
|  |  |  |  |  |
| **Variable** | **n** | **OR^a^** | **95% Confidence interval** | **p-value^b^** |
| **Age (each additional year)** | - | 1.14 | 1.0-1.2 | 0.003 |
| **Unimproved pit latrine** | 385 | 2.6 | 1.5-4.4 | 0.001 |
| **No. of household children aged 1-9 years (each additional child)** | - | 1.3 | 1.0-1.6 | 0.034 |
|  | | | | |
| ^a^ Adjusted Odds Ratio using multivariable two-level mixed effects logistic regression | | |  |  |
| ^b^ p-value from Wald's test^;^ only significant associations (p<0.05) are shown | |  |  |  |
